# A deep learning approach for anterior cruciate ligament rupture localization on knee MR images

**DOI:** 10.3389/fbioe.2022.1024527

**Published:** 2022-09-30

**Authors:** Cheng Qu, Heng Yang, Cong Wang, Chongyang Wang, Mengjie Ying, Zheyi Chen, Kai Yang, Jing Zhang, Kang Li, Dimitris Dimitriou, Tsung-Yuan Tsai, Xudong Liu

**Affiliations:** ^1^ Department of Orthopedics, Shanghai Sixth People’s Hospital Affiliated to Shanghai Jiao Tong University School of Medicine, Shanghai, China; ^2^ College of Electrical Engineering, Sichuan University, Chengdu, China; ^3^ School of Biomedical Engineering and Med-X Research Institute, Shanghai Jiao Tong University, Shanghai, China; ^4^ Department of Radiology, Shanghai Municipal Eighth People’s Hospital, Shanghai, China; ^5^ Department of Radiology, Shanghai Sixth People’s Hospital Affiliated to Shanghai Jiao Tong University School of Medicine, Shanghai, China; ^6^ West China Hospital, Sichuan University, Chengdu, China; ^7^ Department of Orthopedics, Balgrist University Hospital, University of Zürich, Zurich, Switzerland

**Keywords:** artificial intelligence, deep learning, computer-assisted diagnosis, anterior cruciate ligament, localization, primary ACL repair, ACL reconstruction

## Abstract

**Purpose:** To develop and evaluate a deep learning-based method to localize and classify anterior cruciate ligament (ACL) ruptures on knee MR images by using arthroscopy as the reference standard.

**Methods:** We proposed a fully automated ACL rupture localization system to localize and classify ACL ruptures. The classification of ACL ruptures was based on the projection coordinates of the ACL rupture point on the line connecting the center coordinates of the femoral and tibial footprints. The line was divided into three equal parts and the position of the projection coordinates indicated the classification of the ACL ruptures (femoral side, middle and tibial side). In total, 85 patients (mean age: 27; male: 56) who underwent ACL reconstruction surgery under arthroscopy were included. Three clinical readers evaluated the datasets separately and their diagnostic performances were compared with those of the model. The performance metrics included the accuracy, error rate, sensitivity, specificity, precision, and F1-score. A one-way ANOVA was used to evaluate the performance of the convolutional neural networks (CNNs) and clinical readers. Intraclass correlation coefficients (ICC) were used to assess interobserver agreement between the clinical readers.

**Results:** The accuracy of ACL localization was 3.77 ± 2.74 and 4.68 ± 3.92 (mm) for three-dimensional (3D) and two-dimensional (2D) CNNs, respectively. There was no significant difference in the ACL rupture location performance between the 3D and 2D CNNs or among the clinical readers (Accuracy, *p* < 0.01). The 3D CNNs performed best among the five evaluators in classifying the femoral side (sensitivity of 0.86 and specificity of 0.79), middle side (sensitivity of 0.71 and specificity of 0.84) and tibial side ACL rupture (sensitivity of 0.71 and specificity of 0.99), and the overall accuracy for sides classifying of ACL rupture achieved 0.79.

**Conclusion:** The proposed deep learning-based model achieved high diagnostic performances in locating and classifying ACL fractures on knee MR images.

## 1 Introduction

Anterior cruciate ligament (ACL) injuries are common sports-related musculoskeletal diseases (Spindler and Wright, 2008) that increase the risk of developing posttraumatic osteoarthritis and require an early diagnosis and intervention (Wang et al., 2020). In current clinical practice, most orthopedic surgeons will perform ACL reconstruction in patients with ACL injuries (Reijman et al., 2021). However, reconstruction surgery has many disadvantages, such as anterior knee pain (Janani et al., 2020), muscle atrophy (Lindström et al., 2013), and loss of proprioception at the reconstructed surgical site. In addition, native gait kinematics cannot be restored and revision surgery, if necessary, can be difficult due to tunnel widening and malpositioning (Aga et al., 2017; Kraeutler et al., 2017). In the 1970s and 1980s, open primary ACL repair was commonly performed but was eventually abandoned due to poor surgical results and complications (Feagin and Curl, 1976; Taylor et al., 2009). With the development and application of arthroscopy, biotechnology, stronger internal fixation techniques, and more rational postoperative rehabilitation, ACL repair has received renewed attention from orthopedic surgeons (van der List and DiFelice, 2017; DiFelice and van der List, 2018; Mahapatra et al., 2018; Ahmad et al., 2019; Hoogeslag et al., 2019; Murray et al., 2020; Li, 2022). Isolated ACL repair has been reported using various techniques including suture anchor primary ACL repair, internal brace ligament augmentation, bridge-enhanced ACL repair (BEAR), and dynamic intraligamentary stabilization (DIS) methods (Heusdens, 2021). Sherman et al. (Sherman et al., 1991) were the first to classify ACL tears arthroscopically according to both tear location and tissue quality and named it “Sherman classifications” in 1991. More recently, Van der List et al. (van der List and DiFelice, 2016) proposed a treatment algorithm based on the modified Sherman classification and suggested that only proximal ACL tears with good to excellent tissue quality should be repaired. Note that the key question now is how to identify the location and tissue quality of the injured ACL to determine if ACL repair surgery can be performed. Currently, MRI is a non-invasive method that demonstrates excellent sensitivity and specificity for the diagnosis of ACL injuries (Odgaard et al., 2002). Several studies have suggested that MRI may help surgeons to predict the reparability of ACL tears (van der List et al., 2017; van der List and DiFelice, 2018; Mehier et al., 2022). Mehier, C. et al. (Mehier et al., 2022) proposed three classification criteria for ACL tears based on tear location and tissue quality, including MRI Sherman tear location (MSTL), MRI Sherman tissue quality (MSTQ), and simplified MRI Sherman tissue quality (S-MSTQ) classifications. The diagnostic accuracy of the three criteria was 70% (50/71), 52% (15/29), and 90% (26/29), respectively. Interobserver agreement was good for MSTL (*κ* = 0.78) and moderate-to-good for the MSTQ and S-MSTQ classifications (*κ* = 0.44 and 0.63 respectively). Based on the above studies (Sherman et al., 1991; van der List and DiFelice, 2016; van der List et al., 2017; van der List and DiFelice, 2018; Mehier et al., 2022), we focused primarily on the localization of ACL injuries, and we simplified the classification of ACL injury sites to the femoral side, middle and tibial side, with each classification accounting for one-third of the entire ACL.

Deep learning has notable advantages in helping clinicians with limited experience or time in reading MR images and increasing the accuracy of the MR imaging interpretations (Shin et al., 2016). Several previous studies have focused on the application of deep learning for disease diagnoses in medical imaging; the applications include lung adenocarcinoma (Yu et al., 2021), abnormal pulmonary nodules (Sim et al., 2020), and breast masses (Caballo et al., 2020). In the case of diagnosing ACL injuries, previous work has been limited to the use of deep learning methods to detect the presence or absence of ACL injuries (Bien et al., 2018; Liu et al., 2019) and grading the hierarchical severity staging of ACL injuries on knee MR images (Namiri et al., 2020; Awan et al., 2021; Javed Awan et al., 2021). However, deep learning methods have yet to be applied to localizing the ACL rupture.

The main purpose of our study was to develop and evaluate a deep learning-based method to localize and classify ACL ruptures (femoral side, middle and tibial side) (Sherman et al., 1991; van der List and DiFelice, 2016; van der List et al., 2017; van der List and DiFelice, 2018; Mehier et al., 2022) on knee MR images by using arthroscopy as the reference standard.

The remainder of this article is structured as follows. Some of the recent work closely related to this study will be discussed in Section 2. In Section 3, the details of the MRI datasets are presented, and the architecture, implementation details, and performance metrics of the fully automated ACL rupture localization system are presented. The experimental results are analyzed in Section 4. The advantages and limitations of the proposed method are discussed in Section 5. Finally, the conclusion of our study is given in Section 6.

## 2 Recent works

In recent years, various deep learning-based methods have been developed in ACL segmentation and injury assessment. In 2021, Flannery et al. (Flannery et al., 2021) developed an automated intact ACL segmentation model based on 2D U-Net. The reference standard for training the model was the results of segmentation by an experienced (>5 years) physician, and the model was evaluated for anatomical similarity and the accuracy of quantitative metrics (i.e., signal intensity and volume). The model performed well on anatomical performance metrics (Dice coefficient = 0.84, precision = 0.82, and sensitivity = 0.85). The median signal intensities and volumes of the model were not significantly different from the ground truth. Recently, the team used a transfer learning approach to segment the surgically treated ACL automatically (Flannery et al., 2022). Compared with the intact ACL segmentation model, the anatomical performance of the automated segmentation model for surgically treated ACLs was slightly decreased (repairs/grafts: Dice coefficient = 0.80/0.78, precision = 0.79/0.78, sensitivity = 0.82/0.80). There were no significant differences in quantitative metrics between the ground truth and automatic segmentation of surgically treated ACLs. In 2018, Bien et al. (Bien et al., 2018) developed MRNet for detecting ACL tears on knee MR images. Using the labels of three musculoskeletal radiologists with an average of 12 years of experience as a reference standard, researchers evaluated the performance of MRNet and compared it with the performances of nine other physicians (model/physicians: sensitivity = 0.76/0.91, specificity = 0.97/0.93). In addition, the area under the receiver operating characteristic (ROC) curve (AUC) reached 0.82 when validated directly with MRNet on a public dataset from Clinical Hospital Centre Rijeka, Croatia, and improved to 0.91 after retraining. MRNet took less than 30 min to train on and less than 2 min to evaluate the public dataset, indicating that MRNet can improve clinician performance in the interpretation of medical imaging on both internal and external datasets. In 2019, Liu et al. (Liu et al., 2019) proposed a fully automated ACL tear detection system by using two convolutional neural networks (CNNs) to isolate the ACL on knee MR images followed by a classification CNN to detect ACL injuries on the selected image sections. A retrospective study of 350 subjects was conducted to evaluate the sensitivity and specificity of the model and those of the five radiologists in detecting ACL tears using arthroscopy as the reference standard. The overall training time was 11.62 h, while the average time for the model to detect an ACL tear for one subject was 9 s. The sensitivity and specificity of the model at the optimal threshold were 0.96 and 0.96, respectively. In contrast, the sensitivity of the radiologists ranged between 0.96 and 0.98, while the specificity ranged between 0.90 and 0.98. In 2020, Namiri et al. (Namiri et al., 2020) proposed a deep learning-based pipeline to isolate the ACL region of interest (ROI), detect abnormal ACL, and stage lesion severity using three-dimensional (3D) and two-dimensional (2D) CNN, respectively. The overall accuracy of the 3D and 2D CNN in classifying ACL injuries (reconstructed, fully torn, partially torn, and intact ACLs) was 0.89 and 0.92, respectively. In a recent study, Namiri et al. (Astuto et al., 2021) developed a 3D CNN model for full-knee ROI (cartilage, bone marrow, menisci, and ACL) detection and lesion classification. Binary injury sensitivity reported for all tissues was between 0.70 and 0.88, while the specificity ranged from 0.85 to 0.89.

## 3 Materials and methods

### 3.1 MRI datasets

**FIGURE 1:**
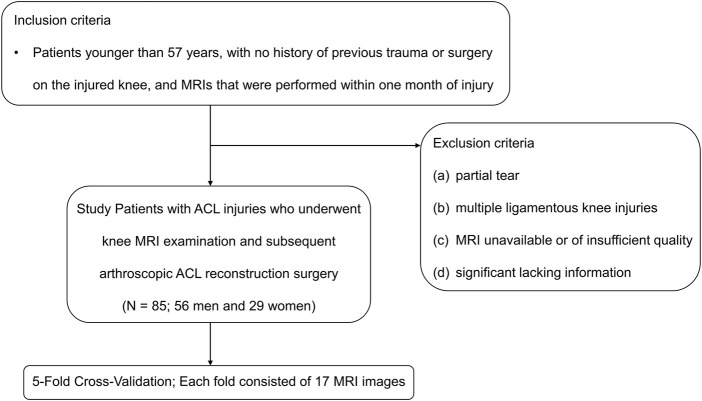
Inclusion and exclusion criteria. ACL, anterior cruciate ligament.

This retrospective study was performed with approval from our institutional internal review boards and ethical committees (Ethics Committee Northeast and Central Switzerland 2018-01410). The MRI datasets were obtained from 85 patients with ACL ruptures (Male: 56, Female: 29) with an average age of 27 (range: 10–57) years who underwent knee MRI examination and subsequent ACL reconstruction surgery under arthroscopy between January 2010 and April 2018 (Figure 1). Inclusion criteria were patients younger than 57 years, with no history of previous trauma or surgery on the injured knee, and MRIs that were performed within 1 month of injury. The patient exclusion criteria were as follows: (a) partial tear; (b) multiple ligamentous knee injuries; (c) MRI unavailable or of insufficient quality; (d) significant lacking information.

All the patients were scanned using a 3.0-T MR Scanner (Achieva; Philips Healthcare, Netherlands). The MRI datasets consisted of sagittal T2-weighted turbo spin-echo and coronal T1–weighted high spatial resolution turbo spin-echo sequences. The detailed imaging parameters of the sequences are summarized in Table 1.

**TABLE 1:** Parameters for the knee MRI sequences used to locate ACL rupture.

Parameter	T1-weighted high spatial resolution turbo spin-echo sequence (min-max, avg)	T2-weighted turbo spin-echo sequence (min-max, avg)
Repetition time (msec)	567-2000 (691)	652-4714.74 (3165.65)
Echo time(s) (msec)	10-15 (14.847)	13-100 (98.74)
Flip angle (degrees)	90	90
Pixel bandwidth (Hz)	110-239 (213.108)	130-293 (288.23)
Echo train length	1-9 (8.08)	1-17
Section thickness (mm)	2.5-3 (2.525)	3-3.3
No. of sections	24-39	22-39
Signal averages	1,2,3	1,2
Acquisition matrix size	320 × 320 - 1600 × 1600	400 × 400 - 1024 × 1024
Reconstruction matrix size	256 × 256	256 × 256
File type	DICOM	DICOM
Bit depth (bit)	16	16

DICOM, digital imaging and communications in medicine.

### 3.2 Fully automated anterior cruciate ligament rupture localization system

In this study, we propose a two-step, coarse-to-fine deep learning-based pipeline to isolate the specific areas that contain ACL in the knee and we locate the ACL rupture site using 2D and 3D convolutions with MR images.

**FIGURE 2:**
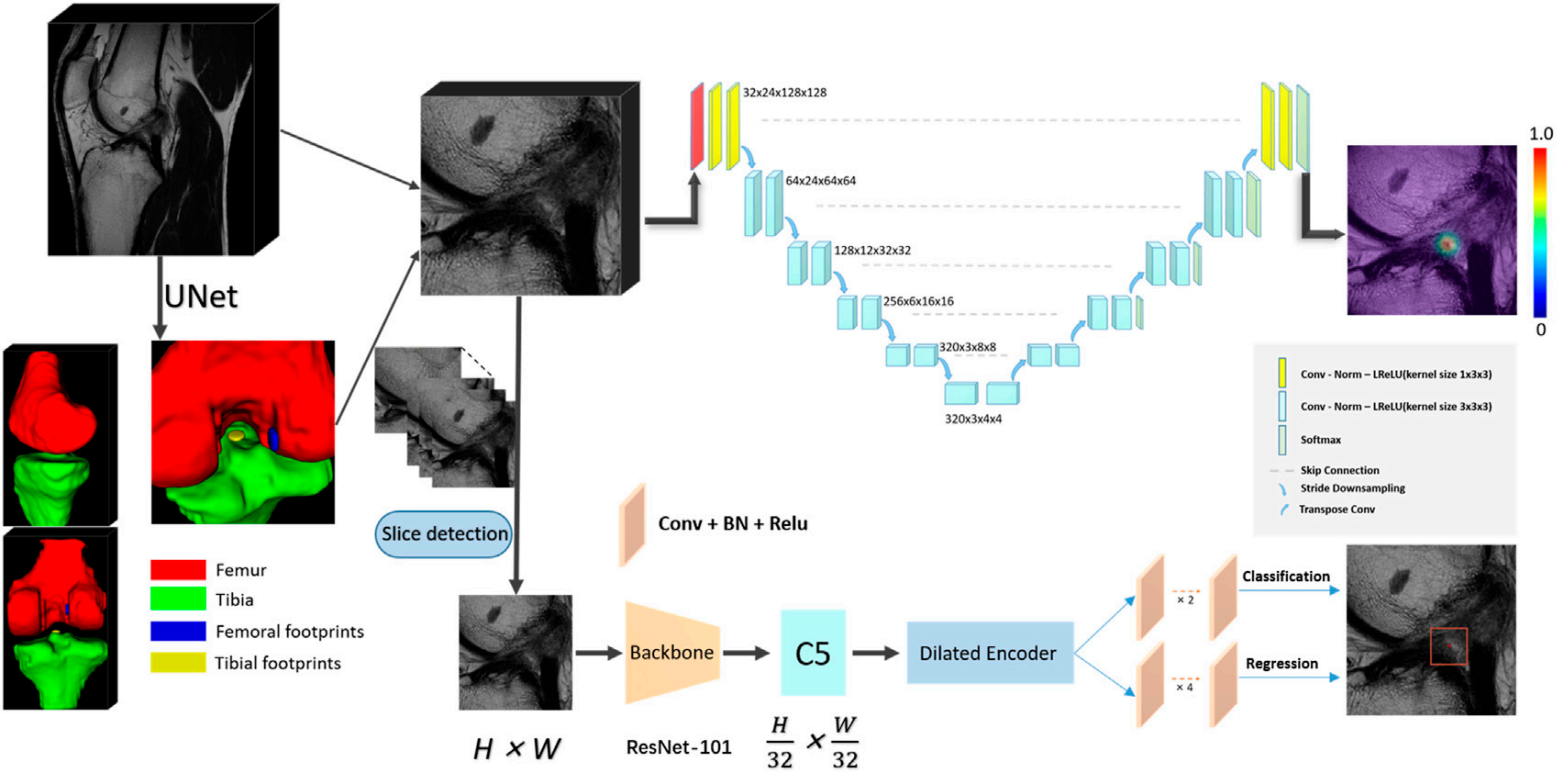
The convolutional neural network (CNN) pipeline for the deep learning-based fully automated ACL rupture localization system. The proposed methods including 2D and 3D CNNs consisted of segmentation and landmark detection network connected in a cascaded fashion to create a fully automated image processing pipeline. ACL, anterior cruciate ligament; BN, batch normalization; Conv, convolution; Norm, normalization; LReLU, Leaky-ReLU; ReLU, rectified-linear activation; 2D, two-dimensional; 3D, three-dimensional.

Our deep learning framework consists of the segmentation network that categorizes the knee into 4 distinct anatomic components and the landmark detection network to localize the centroid of an ACL rupture (Figure 2). The first segmentation network was implemented to approximately narrow the specific areas that contain ACLs; this network was based on a 3D U-Net architecture (Cicek et al., 2016). Based on the position of the femoral footprint and tibial footprint, we cropped the patches containing the ACL from the MR images to eliminate the unnecessary details and used them as input images to the localization CNNs. In the second stage, we compared the localization performance of the CNNs on 2D slices with 3D cropped images. All CNNs were developed through a cascaded approach to create a fully automated processing pipeline. The detailed network structure for the CNNs is summarized in Supplementary Table S1 (Litjens et al., 2017).

#### 3.2.1 2D

**FIGURE 3:**
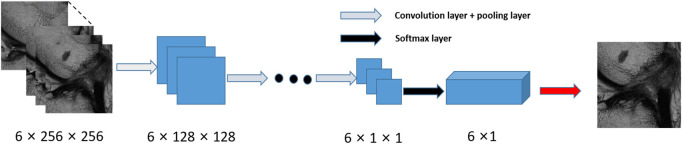
Flow chart for the slice detection network of 2D CNNs.

This scheme consists of two stages, a slice selection and landmark localization. The slice selection network was constructed by a 3D full CNN (Figure 3) with an input size of 6 × 256 × 256, and it had nine sets of convolutional layers and eight pooling layers. The first eight sets of convolutional layers were used to extract features with two 1 × 3 × 3 convolution operations in each layer, while the last convolutional layer was used for reducing the feature dimension to one channel. The image size became 6 × 1 × 1 through eight max pooling layers, which only implemented downsampling on the slice size, followed by a softmax layer, and the network was trained by the cross-entropy loss values between the output vector and standard vector.

The landmark detection stage of our method is mainly based on YOLOF, which is formulated to predict keypoint coordinates by the bounding box center. YOLOF is one of the latest single-level detectors, which only uses the final low resolution feature map C5 to detect objects. We used the ResNet-101 network (Ranjan et al., 2019) as the backbone network in our training phase. Based on the real pixel coordinates (usually decimal) of the rupture point on the axis of the high-resolution slice in the cropped MRI images, we select the integers on both sides of the decimal as the slices (2 slices) where the real rupture point is located. We maintained the slice resolution as 0.25 mm * 0.25 mm in order to use high-precision images that would ensure the accuracy of the results. We utilized random rotation, flipping and elastic transformation to enhance the data and expand our training dataset.

In sum, the selected slice was adopted as the z value of the final coordinate, and the coordinates (x, y) were obtained by the predicted bounding box center. After mapping the obtained pixel coordinates into the physical coordinates of the original MRI image, the automatic localization of the ACL rupture point was completed.

#### 3.2.2 3D

The 3D localization scheme was based on the heatmap regression network, which was adapted from a 3D full resolution nnU-Net (Isensee et al., 2021). The network has an encoder-decoder structure. The encoder is comprised of a sequence of convolution layers with strided convolution downsampling, which compresses the original input volume into low-resolution and highly abstracted feature maps. The decoder has the same structure with a transpose convolution upsampling, which processes the downsampling abstracted feature maps into outputs with the same resolution as the input, in a way that is symmetrical to what is done in the compression layers. The feature maps of the same level are concatenated by a skip connection. All batch normalizations were replaced by group normalizations, and we used the combination of Dice loss and focal loss (Lin et al., 2020) as the loss function to train the model.

In particular, in the training phase, we use a 3D Gaussian function, centered at the manually labeled rupture position, as a probability heatmap. The probability values are multiplied by a constant to scale the maximum to 1 (the groundtruth of the landmark). In the landmark mask, the probability value gradually decreases from 1 at the center position in the voxel range of the Gaussian heatmap distribution (the landmark voxels), and the value of the background voxels is set to zero. Note that we incorporate a false-positive suppression strategy during the training phase to make our model more robust. Specifically, we force the values that are very close to the landmark voxels (e.g., < 2 mm) to be negative rather than zero, so they are regarded as invalid voxels to avoid being calculated in the loss function. Finally, we mark the rupture voxels by using the standard probability threshold of 0.5 and calculate the centroid of the whole region as the output coordinate. The heatmaps were generated using the Matplotlib library (https://matplotlib.org/).

### 3.3 Definition of simplified classification of anterior cruciate ligament injury sites on our deep learning-based model

The ACL rupture was approximately described based on the line connecting the center coordinates of the femoral and tibial footprints. The line was divided into three equal parts to indicate which section (femoral side, middle, and tibial side) the rupture area was located on, while the rupture area was interpreted as the coordinate of the perpendicular foot between the rupture point and the ACL line.

The entire ACL measures approximately 38 mm in length and 11 mm in width (Girgis et al., 1975). According to our simplified classification of ACL injury sites, each section accounts for one-third of the entire ACL, which is approximately 12 mm. Based on the anatomy of ACL (Girgis et al., 1975) and the study of Payer, C. et al. (Payer et al., 2016) on medical image landmark localization, for both 2D and 3D CNNs, a localization failure case occurred when the distance between the ground-truth location and the predicted location was larger than 10 mm.

### 3.4 Implementation

The training and evaluation of our pipeline was done on a desktop computer running a 64-bit Linux operating system with 8 V100 SXM3-32GB GPUs and CUDA version 10.2. All machine learning algorithms were implemented in PyTorch with Python 3.7, and each CNN was trained individually. The model was validated by a fivefold cross-validation. The data were randomly divided up into 5 non-overlapping groups known as folds and each fold consisted of 17 MRI images. One of those folds was retained as the validation set, and the remaining images were used for training. The average accuracy of all the folds was the overall accuracy of our system.

### 3.5 Training and evaluation of the fully automated anterior cruciate ligament rupture localization system

The reference standard for training the segmentation network was the image patch segmentation bounded by a manually labeled femoral footprint and tibial footprint performed on the sagittal T2-weighted sequences of all 85 subjects. The labeling of the femoral footprint and tibial footprint areas was performed by an orthopedic fellow (D.D., with 8 years of labeling experience) using the ITK-SNAP program (https://www.itksnap.org/pmwiki/pmwiki.php). The reference standard for training the localization network was the centroid physical coordinate of the rupture region marked on the MRI of the corresponding patient by an orthopedic fellow (D.D.) using the location of the arthroscopic ACL injury as the reference standard.

### 3.6 Evaluation by clinical readers

To compare the localization accuracy of our pipeline with that of clinical readers, a 3rd-year musculoskeletal clinician [MY (Resident 1)], a 6th-year musculoskeletal clinician [CYW (Resident 2)], and a 6th-year radiologist [ZC (Fellow)] independently reviewed the MR images of all 85 patients. The clinical readers identified the site of the ACL rupture by placing image patches where they believed the ACL rupture occurred on the sagittal T2-weighted MR images using the ITK-SNAP program. Then, the centroid physical coordinates of the manually labeled image patches are calculated and compared with the coordinates predicted by the deep learning-based model to evaluate the localization accuracy and classification performance of the model. All the clinical reviewers had no formal training or calibration courses prior to evaluating the ACL rupture site.

### 3.7 Statistical analysis

All the statistical analyses were calculated using SPSS (Version 26; IBM Corporation, Armonk, NY, United States). The *p* values less than 0.05 were considered statistically significant. Euclidean distances (mean value ±standard deviation, millimeters) between the ground-truth locations and the predicted locations of the landmarks were used to evaluate the algorithm localization accuracy. The localization error rate was defined as the ratio between the number of failure cases and the total samples. Interobserver agreement between two of the three independent blinded clinical readers was assessed using single-measure intraclass correlation coefficients (ICC) with a two-way random-effects model for absolute agreement. The performance statistics for the classification of ACL rupture were reported for sensitivity, specificity, precision, F1-score, and overall accuracy.

$$\begin{array}{c} Sensitivity=TP/\left(TP+FN\right) \end{array}$$ (1)

$$\begin{array}{c} Specificity=TN/\left(TN+FP\right) \end{array}$$ (2)

$$\begin{array}{c} Precision=TP/\left(TP+FP\right) \end{array}$$ (3)

$$\begin{array}{c} F1-score=TP/\left(TP+0.5\;*\;\left(FP+FN\right)\right) \end{array}$$ (4)

$$\begin{array}{c} Overall\;accuracy=\frac{correct\;classifications}{all\;classifications} \end{array}$$ (5)

where TP, TN, FP, and FN are true positive, true negative, false positive, and false negative, respectively. Also, '*' and '/' represent multiplication and division, respectively.

## 4 Results

Compared with models proposed by Bien et al. (Bien et al., 2018) and Liu et al. (Liu et al., 2019), the training time for our pipeline was 60 min, and the average time for the ACL rupture localization system to locate and classify the rupture site for one subject was 1.6 s using the trained networks.

Table 2 compares the accuracy and error rates of the proposed pipeline (both the 2D and 3D methods) with those of the clinical readers. The mean localization accuracies were 4.68 ± 3.92 [standard deviation] (mm) for the 2D method, 3.77 ± 2.74 (mm) for the 3D method, 8.27 ± 4.47 (mm) for Resident 1, 8.34 ± 3.36 (mm) for Resident 2, and 8.00 ± 5.74 (mm) for Fellow. There was no significant difference in ACL rupture location performance between the 3D and 2D CNNs or among the clinical readers (Accuracy, *p* < 0.01). The error rates of the 2D and 3D CNNs were 11% (9/85) and 3.5% (3/85), respectively. In comparison, the error rates of the clinical readers ranged between 31% (28/85) and 40% (34/85). Table 3 shows the ICC values for interobserver agreement between the clinical readers in the localization of ACL ruptures on the same image patches. There was poor to moderate interobserver agreement between the clinical readers, with ICC values between 0.19 and 0.54.

**TABLE 2:** Accuracy and error rate of clinical residents, musculoskeletal radiology fellow, 2D CNNs, and 3D CNNs in localization of ACL ruptures.

	Accuracy* (mm)	Error rate (%)
2D method	4.68 ± 3.92	11 (9/85)
3D method	3.77 ± 2.74	3.5 (3/85)
Resident 1	8.27 ± 4.47^a^ ^,^ ^b^	31 (28/85)
Resident 2	8.34 ± 3.36^a^ ^,^ ^b^	40 (34/85)
Fellow	8.00 ± 5.74^a^ ^,^ ^b^	31 (28/85)
*p* value	<0.01	

*Euclidean distances (mean value ±standard deviation) used to evaluate the localization accuracy.

^a,b^ *p* < 0.01 vs. 2D method group. *p* < 0.01 vs. 3D method group.

ACL, anterior cruciate ligament; CNNs, convolutional neural networks; 2D, two-dimensional; 3D, three-dimensional.

**TABLE 3:** Intraclass correlation coefficients (ICC) for Interobserver Agreement between the Clinical Readers in Localization of ACL Ruptures.

Reader	Resident 1	Resident 2	Fellow
Resident 1	NA	0.54 (0.37, 0.68)	0.32 (0.12, 0.50)
Resident 2	0.54 (0.37, 0.68)	NA	0.19 (−0.03, 0.38)
Fellow	0.32 (0.12, 0.50)	0.19 (−0.03, 0.38)	NA

Data are ICC values, with 95% confidence intervals in parentheses. NA, not applicable.

Tables 4, 5 show the confusion matrices and sensitivity and also the specificity, precision, F1-score, and overall system accuracy values for the clinical readers, and also the 2D and 3D CNNs for evaluating the side classification performance on ACL ruptures on the image patches in all 85 MR datasets. The confusion matrix results for the ACL injury classification corresponding to each evaluator in Table 4 show that the 3D CNNs had the highest performance on ACL rupture classifications. As shown in Table 5, both models performed better than the clinical readers in describing the location of ACL ruptures. The 3D CNNs performed best among the five evaluators in classifying the femoral side (sensitivity of 0.86 and specificity of 0.79), middle side (sensitivity of 0.71 and specificity of 0.84), and tibial side ACL rupture (sensitivity of 0.71 and specificity of 0.99). While the overall accuracy of clinical readers ranged between 0.42 and 0.56, the overall accuracy of the ACL localization system for the 3D and 2D CNNs was 0.79 and 0.61, respectively.

**TABLE 4:** Confusion matrices for the clinical residents, musculoskeletal radiology fellow, 2D CNNs, and 3D CNNs for performance in sides classifying of ACL rupture on the image patches.

Predict truth	Femoral side	Middle	Tibial side
Resident1			
femoral side	8	0	0
middle	30	30	4
tibial side	5	5	3
Resident2			
femoral side	1	0	0
middle	40	32	4
tibial side	1	3	3
Fellow			
femoral side	32	10	3
middle	6	13	1
tibial side	5	12	3
2D CNNs			
femoral side	28	10	1
middle	13	23	5
tibial side	2	2	1
3D CNNs			
femoral side	37	9	0
middle	6	25	2
tibial side	0	1	5

ACL, anterior cruciate ligament; CNNs, convolutional neural networks; 2D, two-dimensional; 3D, three-dimensional.

**TABLE 5:** Sensitivity, specificity, precision, F1-score, and overall accuracy for clinical residents, musculoskeletal radiology fellow, 2D CNNs, and 3D CNNs for performance in sides classifying of ACL rupture on the image patches.

	Position class	Sensitivity	Specificity	Precision	F1-score	Overall accuracy
3D CNNs	Femoral side	0.86	0.79	0.80	0.83	0.79
	Middle	0.71	0.84	0.76	0.74	
	Tibial side	0.71	0.99	0.83	0.77	
2D CNNs	Femoral side	0.65	0.74	0.72	0.68	0.61
	Middle	0.66	0.64	0.56	0.61	
	Tibial side	0.14	0.95	0.2	0.17	
Resident 1	Femoral side	0.19	1	1	0.31	0.48
	Middle	0.86	0.32	0.47	0.61	
	Tibial side	0.43	0.87	0.23	0.3	
Resident 2	Femoral side	0.02	1	1	0.05	0.42
	Middle	0.91	0.10	0.42	0.58	
	Tibial side	0.43	0.95	0.43	0.43	
Fellow	Femoral side	0.74	0.69	0.71	0.73	0.56
	Middle	0.37	0.86	0.65	0.47	
	Tibial side	0.43	0.78	0.15	0.22	

ACL, anterior cruciate ligament; CNNs, convolutional neural networks; 2D, two-dimensional; 3D, three-dimensional.

**FIGURE 4:**
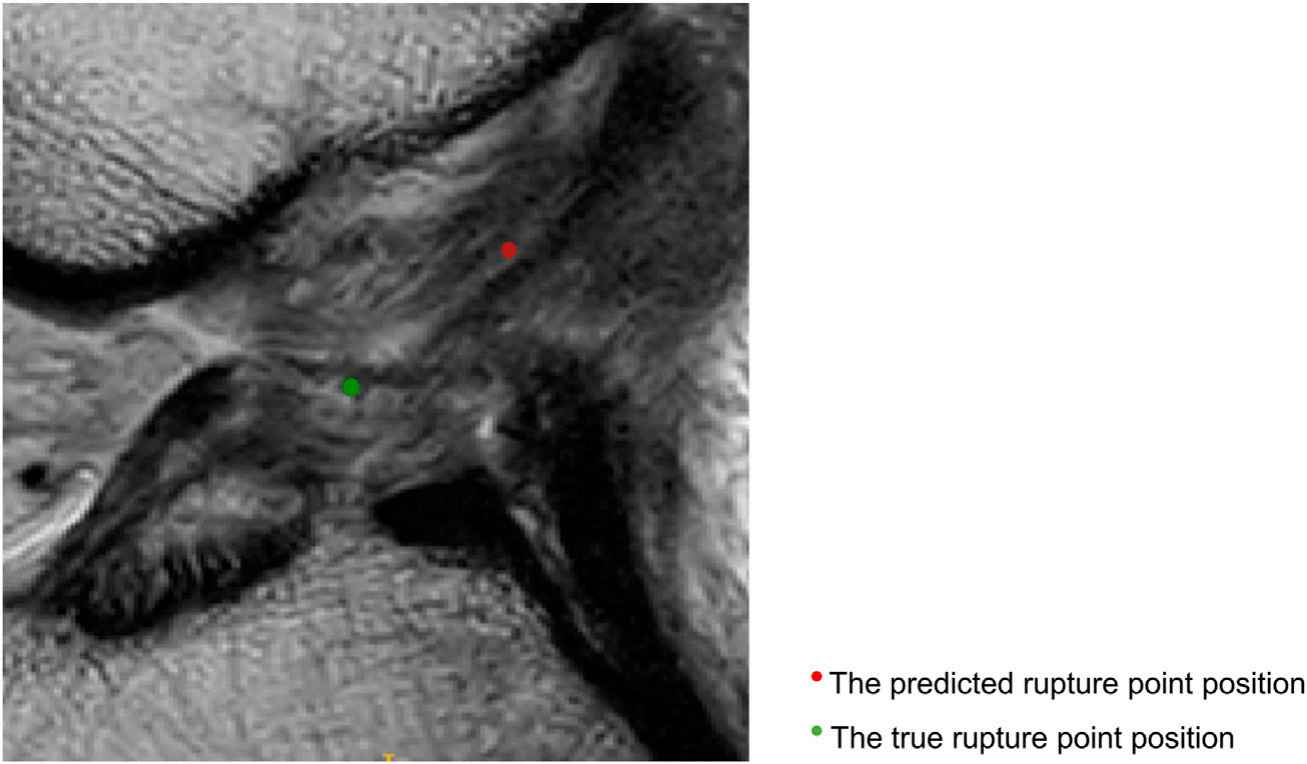
Sagittal views of the cropped MR image, mislocalization and false classification. The predicted rupture point is marked by **red** circle, while the true rupture point is **green**. The deep learning pipeline outputs incorrect localization results due to the Euclidean distance between the true and predicted rupture point locations being greater than 10 mm, which exceeds the maximum error threshold we set. A mislocalization resulted in a false classification. The true part of the rupture is the middle side, but the prediction is femoral side.

Figure 4 displays sagittal views of the cropped knee MR image, which were processed by the deep learning model for mislocalization and false classification. The true part of the rupture is on the middle side but the model outputs a classification result on the femoral side. The deep learning pipeline outputs incorrect localization results due to the Euclidean distance between the true and predicted rupture point locations being greater than 10 mm, which exceeds the maximum error threshold we set. Based on our model, the results of ACL rupture classification are directly related to the accuracy of its rupture localization, and incorrect localization leads to incorrect classification.

**FIGURE 5:**
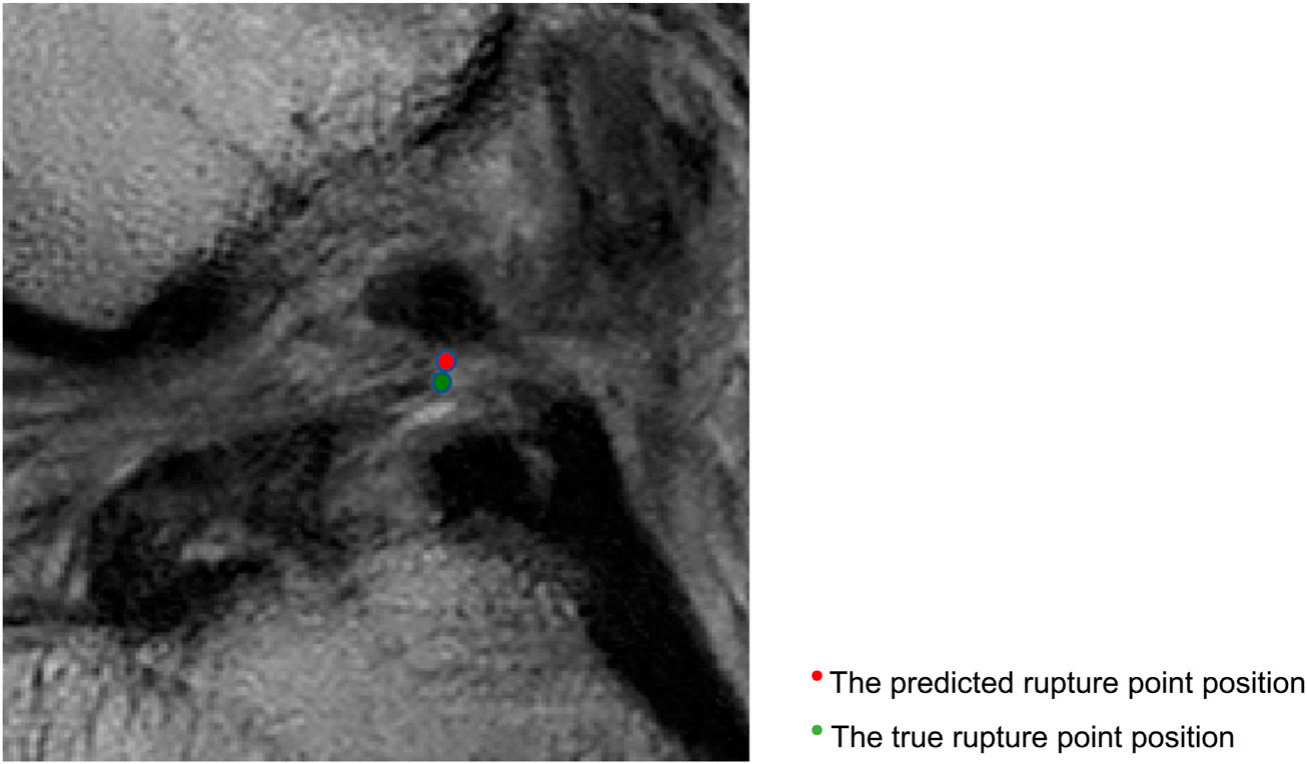
Sagittal views of the cropped MR image, correct localization and classification. The predicted rupture point is marked by **red** circle, while the true rupture point is **green**. The model predicted a correct localization, and the system shows a correct classification (the middle side).

Figure 5 shows that the predicted rupture point location is very close to the true rupture point location and the Euclidean distance between them is within the set error range. The deep learning model is able to correctly locate the ACL rupture point and therefore outputs the correct classification.

## 5 Discussion

Our study describes a fully automated ACL rupture localization system utilizing a segmentation network adapted from 3D U-Net (Cicek et al., 2016) for approximately narrowing the specific areas that contain an ACL. This is followed by a second landmark detection network based on the YOLOF (for the 2D model) and 3D full resolution nnU-Net (Isensee et al., 2021) (for the 3D model) with several modifications to localize the ACL rupture within the cropping patches that contain the ACL rupture region of interest according to the coordinate. The 3D CNNs achieved the highest performance among all the models and clinicians, with a localization accuracy reaching 3.77 ± 2.74 (mm). The error rate and the overall system accuracy were 3.5% (3/85) and 79%, respectively. In addition, the 3D CNNs performed best among the five evaluators in classifying the femoral side (F1-score: 0.83), middle side (F1-score: 0.74), and tibial side ACL rupture (F1-score: 0.77).

Previous work using deep learning methods has been limited to detecting the presence or absence of ACL ruptures or triaging the lesion severity of ACL injuries on knee MR images. Bien et al. (Bien et al., 2018) made predictions from three series types of knee MRIs to train different MRNets with a pretrained AlexNet, and the experimental results showed a 0.911 AUC, 0.968 specificity, and 0.759 sensitivity for ACL tears. Namiri et al. (Namiri et al., 2020) created a deep learning model to predict four lesion severities for the ACL, used V-Net to segment the knee and determined the ACL boundaries of the original input MRI. Then, the cropped images were tested on the 2D and 3D CNNs, which detected reconstructed, fully torn, partially torn and intact ACLs. The 2D and 3D CNNs achieved high overall accuracies of 92% and 89%, respectively. Most recently, Awan et al. (Javed Awan et al., 2021) trained a customized ResNet-14 architecture utlilizing class balancing and data augmentation, which performed at an average accuracy of 92% for three classes. The results showed that the AUC was 0.980 for healthy ACLs, 0.970 for partially torn ACLs and 0.999 for fully torn ACLs. In contrast to the work described above, our pipeline has many advantages. Our pipeline localizes and classifies ACL ruptures on knee MR images, which can help clinicians roughly determine whether a patient has a potential for ACL repair based on the results of ACL injury classification. Based on the ACL injury treatment algorithm proposed by Van der List et al. (van der List and DiFelice, 2016; van der List et al., 2017), we believe that proximal ACL injuries (femoral side) have the potential for ACL repair surgery whereas ACL reconstruction surgery is recommended for injuries near the middle and tibial sides. In addition, our localization system is not influenced by human factors. The interobserver agreement between clinicians in our study did not perform very well (ICC range between 0.19 and 0.54), which may be due to inexperience, distraction, and different interpretations of MRI by clinicians with different specialties. Our pipeline avoids these problems by using arthroscopy as a reference standard and labeling the location of the ACL injury on the corresponding MR images. Furthermore, both CNNs and clinical readers localized ACL rupture within a set threshold (10 mm), but CNNs performed better than clinicians in localization (CNNs/clinicians: 3.77–4.68 mm/8.00–8.34 mm). With accurate localization of ACL injuries, our system also allows the surgeon to adjust the range of ACL injury classification to suit the actual situation.

Our study had several limitations. First, our dataset has a small sample size which only allows for the process of data cross-validation, and more data are needed to verify the reliability of our system. Second, proton density-weighted MR sequences are considered to be commonly used to evaluate knee injuries. MR data in our study are sagittal T2-weighted and coronal T1–weighted MR sequences, and more sequences need to be added to train the localization system to make the results more reliable. In addition, given the fair interobserver agreement among clinicians, we need more experienced clinicians to join the evaluators to calculate ACL injury localization accuracy and classification reliability. Finally, we can include the negative control group in which there is no ACL rupture in the development of the deep learning pipeline, which may be of greater translational and applied value to clinical scenarios.

## 6 Conclusion

In conclusion, our pipeline was found to be more accurate in locating and classifying ACL ruptures (femoral side, middle, and tibial side) than clinicians with varying levels of experience, which may help clinicians determine whether an ACL injured patient has the potential for ACL repair based on the classification results.

## Data Availability

The raw data supporting the conclusion of this article will be made available by the authors, without undue reservation.
