# Supplementary material for: A deep learning approach for anterior cruciate ligament rupture localization on knee MR images
Source: Front Bioeng Biotechnol. 2022 Sep 30;10:1024527. doi: 10.3389/fbioe.2022.1024527 (PMC9561886; doi:10.3389/fbioe.2022.1024527)
Supplement: Supplementary file 1 [file Table1.DOCX]

Supplementary Material

**Supplementary Table 1** Detailed Network Structure for the Segmentation, Slice Detection, and Localization CNNs of the Deep Learning-based Fully Automated ACL Rupture Localization System.

| Segmentation Network | Slice Detection Network | Localization Network |
| --- | --- | --- |
| Input (24×128×128 image patch) | Input (6×256×256 image patch) | Conv2D (64 7× 7 filters, stripe size 2), BN, ReLU |
| Conv3D (32 1× 3× 3 filters), GN, LReLU | Conv3D (32 1× 3× 3 filters), BN, ReLU | MaxPool (window size 3 ×3, stripe size 2) |
| Conv3D (32 1× 3× 3 filters), GN, LReLU | Conv3D (32 1× 3× 3 filters), BN, ReLU | Resblock: { Conv2D (64 1× 1 filters), BN, ReLU; Conv2D (64 3× 3 filters), BN, ReLU; Conv2D (256 1× 1 filters), BN, ReLU}×3 |
| Conv3D (64 3× 3× 3 filters, 1× 2× 2 stride), GN, LReLU | MaxPool (window size 2 ×2, stripe size 2) | Resblock: { Conv2D (128 1× 1 filters), BN, ReLU; Conv2D (128 3× 3 filters), BN, ReLU; Conv2D (512 1× 1 filters), BN, ReLU}×4 |
| Conv3D (64 3× 3× 3 filters), GN, LReLU | Conv3D (64 1× 3× 3 filters), BN, ReLU | Resblock: { Conv2D (256 1× 1 filters), BN, ReLU; Conv2D (256 3× 3 filters), BN, ReLU; Conv2D (1024 1× 1 filters), BN, ReLU}×23 |
| Conv3D (128 3× 3× 3 filters, 2× 2× 2 stride), GN, LReLU | Conv3D (64 1× 3× 3 filters), BN, ReLU | Resblock: { Conv2D (512 1× 1 filters), BN, ReLU; Conv2D (512 3× 3 filters), BN, ReLU; Conv2D (2048 1× 1 filters), BN, ReLU}×4 |
| Conv3D (128 3× 3× 3 filters), GN, LReLU | MaxPool (window size 2 ×2, stripe size 2) | Conv2D (512 1× 1 filters), BN, ReLU |
| Conv3D (256 3× 3× 3 filters, 2× 2× 2 stride), GN, LReLU | Conv3D (128 1× 3× 3 filters), BN, ReLU | Conv2D (512 3× 3 filters), BN, ReLU |
| Conv3D (256 3× 3× 3 filters), GN, LReLU | Conv3D (128 1× 3× 3 filters), BN, ReLU | Resblock: { Conv2D (128 1× 1 filters), BN, ReLU; Conv2D (128 3× 3 filters), BN, ReLU; Conv2D (512 1× 1 filters), BN, ReLU}×4 |
| Conv3D (320 3× 3× 3 filters, 2× 2× 2 stride), GN, LReLU | 6×[MaxPool (window size 2 ×2, stripe size 2) | Conv2D (512 3× 3 filters), BN, ReLU |
| Conv3D (320 3× 3× 3 filters), GN, LReLU | Conv3D (256 1× 3× 3 filters), BN, ReLU | Conv2D (512 3× 3 filters), BN, ReLU |
| Conv3D (320 3× 3× 3 filters, 1× 2× 2 stride), GN, LReLU | Conv3D (256 1× 3× 3 filters), BN, ReLU] | Conv2D (512 3× 3 filters), BN, ReLU |
| Conv3D (320 3× 3× 3 filters), GN, LReLU | Conv3D (1 1× 1× 1 filters), BN, ReLU | Conv2D (512 3× 3 filters), BN, ReLU |
| ConvTranspose3d (320 3× 3× 3 filters, stripe size 2), GN, LReLU | SoftMax (2 classes) | Conv2D (4 3× 3 filters), BN, ReLU (2 corner x,y coordinate) |
| Conv3D (320 3× 3× 3 filters), GN, LReLU | … | … |
| ConvTranspose3d (256 3× 3× 3 filters, stripe size 2), GN, LReLU | … | … |
| Conv3D (256 3× 3× 3 filters), GN, LReLU | … | … |
| ConvTranspose3d (128 3× 3× 3 filters, stripe size 2), GN, LReLU | … | … |
| Conv3D (128 3× 3× 3 filters), GN, LReLU | … | … |
| ConvTranspose3d (64 3× 3× 3 filters, stripe size 2), GN, LReLU | … | … |
| Conv3D (64 3× 3× 3 filters), GN, LReLU | … | … |
| ConvTranspose3d (32 1× 3× 3 filters, stripe size 2), GN, LReLU | … | … |
| Conv3D (32 1× 3× 3 filters), GN, LReLU | … | … |
| Conv3D (1 1× 1× 1 filters), SoftMax (5 classes for the segmentation,2 classes for the landmark heatmap) | … | … |

Note.—The convolutional neural networks (CNNs) were built by using the listed layers from the top to the bottom. Definitions of the different network structures and layers of a CNN have been described in a previously published review article (Litjens et al., 2017).

ACL, anterior cruciate ligament; BN, batch normalization; Conv2D, two-dimensional convolution; Conv3D, three-dimensional convolution; ConvTranspose3d, transposed three-dimensional convolution; GN, Group normalization; LReLU, Leaky-ReLU; MaxPool, maximum pooling; ReLU = rectified linear activation.
